# Development of an Automated and Sensitive Microfluidic Device for Capturing and Characterizing Circulating Tumor Cells (CTCs) from Clinical Blood Samples

**DOI:** 10.1371/journal.pone.0147400

**Published:** 2016-01-25

**Authors:** Priya Gogoi, Saedeh Sepehri, Yi Zhou, Michael A. Gorin, Carmela Paolillo, Ettore Capoluongo, Kyle Gleason, Austin Payne, Brian Boniface, Massimo Cristofanilli, Todd M. Morgan, Paolo Fortina, Kenneth J. Pienta, Kalyan Handique, Yixin Wang

**Affiliations:** 1 Celsee Diagnostics, 46701 Commerce Center Drive, Plymouth, Michigan, United States of America; 2 The James Buchanan Brady Urological Institute and Department of Urology, Johns Hopkins University School of Medicine, Baltimore, Maryland, United States of America; 3 Sidney Kimmel Cancer Center, Department of Cancer Biology, Thomas Jefferson University, Philadelphia, Pennsylvania, United States of America; 4 Laboratory of Molecular Biology, Department of Laboratory Medicine, University Hospital “A. Gemelli”, Rome, Italy; 5 Division of Hematology/Oncology, Robert Lurie Cancer Center, Northwestern University, Chicago, Illinois, United States of America; 6 Comprehensive Cancer Center, University of Michigan, Ann Arbor, Michigan, United States of America; 7 Department of Molecular Medicine, University of Rome “Sapienza”, Rome, Italy; The Ohio State University, UNITED STATES

## Abstract

Current analysis of circulating tumor cells (CTCs) is hindered by sub-optimal sensitivity and specificity of devices or assays as well as lack of capability of characterization of CTCs with clinical biomarkers. Here, we validate a novel technology to enrich and characterize CTCs from blood samples of patients with metastatic breast, prostate and colorectal cancers using a microfluidic chip which is processed by using an automated staining and scanning system from sample preparation to image processing. The Celsee system allowed for the detection of CTCs with apparent high sensitivity and specificity (94% sensitivity and 100% specificity). Moreover, the system facilitated rapid capture of CTCs from blood samples and also allowed for downstream characterization of the captured cells by immunohistochemistry, DNA and mRNA fluorescence in-situ hybridization (FISH). In a subset of patients with prostate cancer we compared the technology with a FDA-approved CTC device, CellSearch and found a higher degree of sensitivity with the Celsee instrument. In conclusion, the integrated Celsee system represents a promising CTC technology for enumeration and molecular characterization.

## Introduction

Cancer metastasis occurs when tumor cells disassociate from the primary tumor, enter the circulation, and migrate to distant organs through the peripheral blood stream or lymphatic drainage [1, 2]. Circulating cells with the characteristics of tumor cells of epithelial origin, commonly termed circulating tumor cells (CTCs), are frequently present in the blood and bone marrow of patients with cancers of the breast, prostate and colon [3–9]. The cells have been detected not only in patients with metastatic cancer, but also those with clinically localized disease [1, 10–11]. Although CTCs are present in small numbers, present in concentrations as low as one cell per 100 million to 1 billion blood cells, molecular characterization of even a small number of CTCs may provide an improved understanding of the metastatic cascade, help with risk stratification and enable therapeutic selection and monitoring of progression for patients undergoing treatment [3, 4, 8, 9].

In clinical practice, analysis of CTCs can provide a minimally-invasive approach for tumor characterization [5, 6, 7]. A variety of technologies have been developed to improve detection and capture of CTCs from peripheral blood. These methods include immunomagnetic bead separation using antibodies targeting epithelial cell-surface antigens, cell sorting using flow cytometry, filtration-based size separation, density gradient centrifugation, and high-throughput imaging [10–24]. At present, Epithelial Cell Adhesion Molecule (EpCAM) is the most commonly used epithelial biomarker used for the detection of CTCs. In fact, this protein is required for the capture of CTCs using the only FDA-approved clinical CTC platform, CellSearch (Veridex LLC. Raritan, NJ) [5, 6]. A key limitation of this approach, however, is the potential inability to detect CTCs with low or absent EpCAM expression [25].

We previously reported a novel microfluidic chip-based device to capture and analyze CTCs [26]. In the work we used the Celsee device to investigate normal donor blood samples spiked with different cancer cells including MCF7, MDA-MB-231 and SKBR3, as well as a panel of cancer biomarkers. We were able to show that the device can capture cells in a range of 20–2,000 with high reproducibility. The capturing efficiency of the cells is greater than 80% with a minimum background of leukocyte contamination in the captured cell population. Furthermore, it captured both epithelial cancer cells such as MCF7 and SKBR3 and mesenchymal cells such as MDA-MB-231. The previous study served as the foundation for the current studies in this manuscript. The Celsee PREP 400^™^ system (Celsee Diagnostics, Plymouth, MI) represents an automated system of this microfluidic device and contains a parallel network of 4 microfluidic chips each of which has approximately 56,320 capture chambers [27]. Based on differences in cell size and deformability, each chamber ensures that small blood cells such as red blood cells and most leukocytes escape while larger cancer cells are trapped and isolated in the chamber. Because the device utilizes a label-free approach, the downstream of identification of individual CTCs can utilize a wide array of antibodies and DNA/RNA-based probes. In addition, since target cancer cells are segregated into individual chambers, this platform limits the potential for leukocyte contamination.

In this study, we report the results of CTC enrichment and analysis from blood samples of patients with metastatic breast, prostate, and colorectal cancer using the automated Celsee PREP 400 system. Our data suggest that CTCs can be captured efficiently in these patients by size filtration, highlighting the potential clinical value of this technology.

## Materials and Methods

### Ethics statement and clinical sample preparation

This study was approved by respective institutional review boards (IRB) at Thomas Jefferson University, Johns Hopkins University School of Medicine and University of Michigan. Informed and written consent was obtained from all patients. Blood samples from patients with metastatic prostate cancer were obtained from Johns Hopkins University and University of Michigan. Patients were monitored closely for disease progression using serum PSA and imaging. Eighteen of the prostate cancer samples were also tested by using both Celsee and CellSearch systems. This group has a mean PSA of 109.3 ng/ml (median = 55.5 ng/ml). Patient samples from Thomas Jefferson University were women with metastatic breast cancer. Among the patients, 41 have known status on ER (25 ER+, 16 ER-); 40 have known status on PR (17 PR+, 23 PR-); and 37 had known status on HER-2 (7 HER-2+, 30 HER-2-). Following informed consent, blood samples from healthy donors and patients with metastatic breast, prostate and colorectal cancer were acquired. The samples were collected from 3 institutions, Thomas Jefferson University, Johns Hopkins University School of Medicine and University of Michigan. Blood samples were collected, stored and transported in CellSave tubes (Veridex, Raritan, NJ), 10 ml BCT Cell-free DNA tubes (Streck, La Vista, NE) or EDTA-coated vacutainer tubes (Becton-Dickinson, Franklin Lakes, NJ), and shipped to Celsee Diagnostics for processing. Samples collected in either CellSave tubes or BCT Cell-free DNA tubes were used for CTC capturing and immunostaining. Samples collected in EDTA-coated tubes were used for CTC capturing and DNA and mRNA FISH analysis. All the tubes have no effect on and work equally well for immunostaining. However, only EDTA-coated tubes work fine for FISH assays because the other tubes contain fixatives that damage nucleic acids.

### Microfluidic device

**Fig 1:**
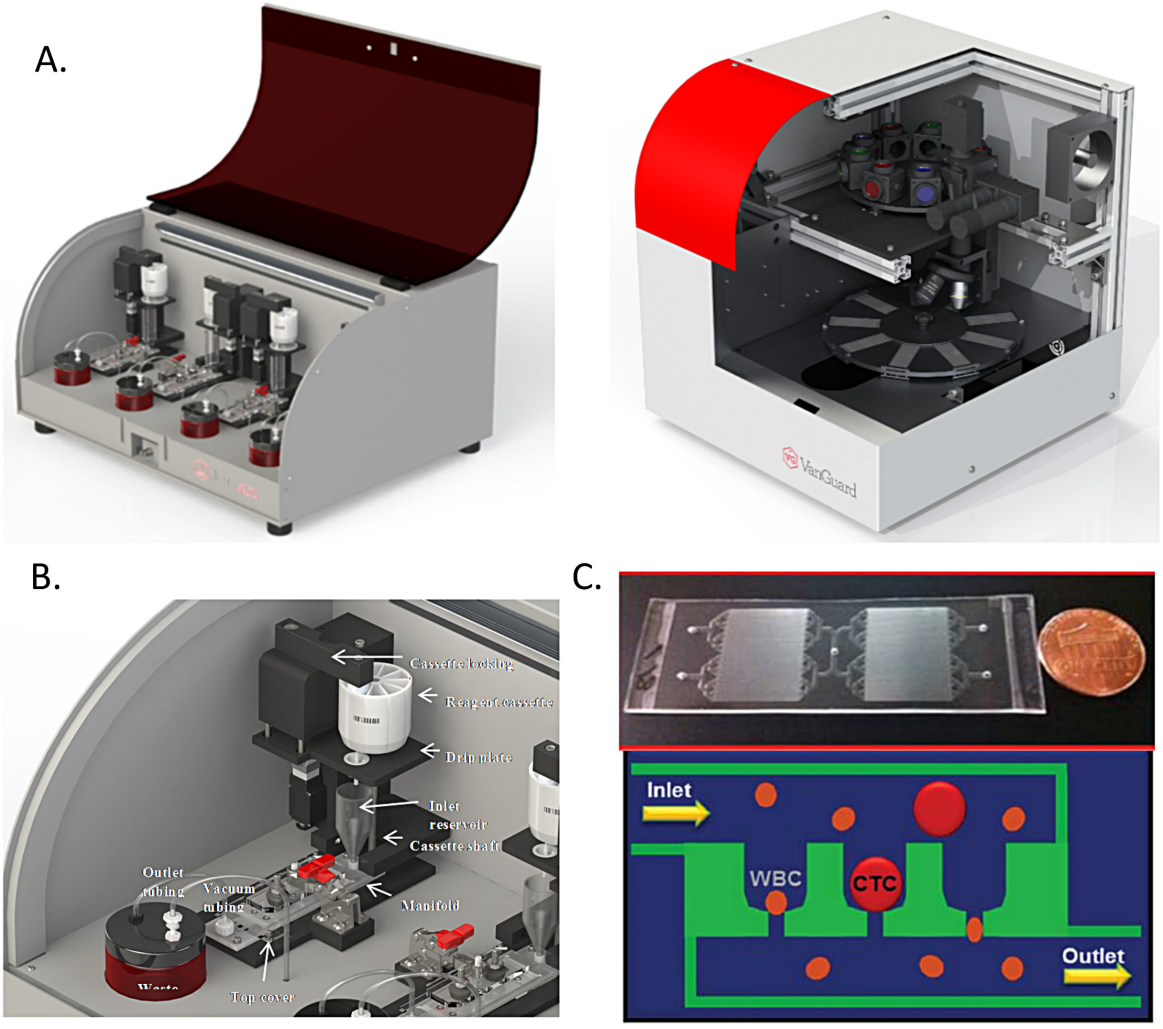
Overview of the Celsee Diagnostics CTC system. (A) Celsee PREP 400 system (left) for blood sample processing and Celsee Analyzer (right) for image analysis. (B) Design of the Celsee PREP 400 microfluidic flow. (C) Design of microfluidic chip (top) and mechanism of capturing CTCs with inlet and outlet for pumping blood samples and reagents through the device (bottom). CTCs are captured in microchannel chambers of the chip while red blood cells and most leukocytes go through the microchannel chambers into the outlet to achieve separation.

The integrated CTC analysis system consists of two instruments: Celsee PREP^™^ 400 sample processing system and Celsee Analyzer^™^ imaging station (Fig 1A). The Celsee PREP^™^ 400 system captures, enumerates and performs single cell analysis of CTCs directly from blood samples (Fig 1B, top). At the core of the Celsee PREP^™^ is a novel microfluidic slide (Fig 1B, bottom) that consists of 56,320 capture chambers to ensure isolation of CTC in individual compartments enabling cellular and genomic analysis to be performed at a single cell level [26]. The microfluidic slide consists of a fluidic network (~75 μm deep) leading to multiple cell trapping chambers (20 x 20 x 30 μm) with individual pore channels (7.5 μm in width x 8 μm in depth and 10 μm in length). This process uses standard micro-fabrication techniques. The size-based filtration for CTC capturing is described in Fig 1C. The Celsee PREP^™^ microfluidic slide captures and isolates rare CTCs from blood samples based on their size and deformability differences from blood cells with a very high efficiency (>85%) [26]. The label free capture process allows researchers to customize assays based on the size of the cells they are seeking to isolate. Furthermore, it also ensures contaminating leukocytes trapped in separate chambers for subsequent identification using CD45 counterstaining. The Celsee PREP^™^ 400 system can run four blood samples on four microfluidic slides simultaneously (Fig 1A). Each lane is set up to process one slide with a top slot for the reagent cassette, and this automatically dispenses liquid into the inlet funnel. The inlet funnel leads to the microfluidic slide through a fluidic manifold. Reagents flow through the slide to a disposable waste jar aided by a vacuum pump (Fig 1B). All materials directly contacting patient samples is disposable: the Celsee PREP microfluidic slide, fluidic manifold, waste jars and inlet funnels (Fig 1C). In addition, the reagent cassette is also disposable. When the samples are loaded into the inlet funnel, a user initiates blood processing and staining. Once the slides are processed, they are viewed with the Celsee Analyzer imaging station.

### Automated Imaging System

The Celsee Analyzer^™^ imaging station automatically captures bright field or fluorescence images of cells while its software allows specific analysis and reporting of the results. It is an automatic whole slide scanner that is comprised of the following components: an optical focusing system, a CCD camera, a Z-focusing system, an XY-translational stage, bright field illumination, a multi-color fluorescence excitation and emission filter system, a microprocessor control board, and a computer. The user can upload up to 8 slides for processing and can view images in up to 6 fluorescent colors. Celsee Analyzer scans the entire surface of the microfluidic slide with a series of fluorescence filters that are defined for a given assay and acquires images. A standard assay includes capture of images using filters for DAPI (nucleus), Alexafluor 488 (pan cytokeratin [PanCK], an epithelial marker) and Alexafluor 594 (CD45, a leukocyte marker). Additionally the user can capture data with filters for Cy3, Cy5 or PE and APC. After data acquisition is completed, the images are analyzed for any event where DAPI and cytokeratin are within a specified space in the slide, i.e., indicating the possible presence of a cell with a nucleus that expresses cytokeratin. Images from each fluorescent color, as well as a composite image are presented to the user in a gallery for CTC calls. A cell is classified as a CTC when it is DAPI+, PanCK+ and CD45-. A check mark is placed by the operator next to the composite images and the software tallies all the checked boxes to obtain a CTC count in the sample.

### Sample processing

The blood samples collected in the BCT Cell Free DNA tubes and CellSave tubes are processed within 72 hours while the blood sample collected in the EDTA-coated vacutainer tubes are processed within 24 hours of collection. The first step of blood preparation begins with adding 2 ml pre-treatment buffer of 0.8% paraformaldyde (PFA) to 2 ml blood. The solution is then mixed and incubated for 10 minutes in order to pre-fix the cells. This brief partial fixation step of the cells with a low concentration of the fixative ensure that the cells will have appropriate deformability while passing through the microfluidic slide. After the incubation, 2 ml of wash buffer (1X PBS) are added to the sample and the sample is loaded to the inlet funnel of one of the 4 lanes of the Celsee PREP 400 instrument. The rest of the steps are automated. Following the cell capturing, the primary antibody cocktail, Anti-PanCK (Clone C11, BioLegend, CA) and Anti-CD45 (Clone F10-89-4, AbD Serotec, CA) is added and incubated for 45 minutes, followed by a washing and an incubation with secondary antibody cocktail of Alexaflour 488 and Alexaflour 594 (Life Technologies, CA) for 30 minutes. Owing to their bigger size compared to white blood cells, cancer cells are captured by micro-chambers and the remaining solution containing red blood cells and the majority of leukocytes are collected by the outlet reservoir after passing through the pore chambers. A background level of larger leukocytes such as monocytes are also trapped by the micro-chambers but are distinguished by their surface markers in the subsequent analysis. In selected samples, a second 2 ml of blood is processed and the captured CTCs are stained with monoclonal Anti-vimentin (Rabbit monoclonal EPR3776, Life Technologies, CA). Selected prostate samples are also stained with monoclonal Anti-PSA (Life Technologies, CA).

### Microscope imaging, enumeration and Immunofluorescence staining of CTCs

Captured cells were imaged using the Celsee Analyzer system. All images were taken with the same exposure time and conditions in order to compare the relative fluorescence intensity. CTC enumeration following antibody labeling and imaging capturing was performed manually. Nucleated PanCK+/CD45- cells were identified as CTCs. Positive and negative controls with cultured cancer cell lines for antibody performance and staining were included in each experiment.

### DNA Fluorescence in-situ hybridization (FISH)

Two milliliters of blood samples from metastatic breast cancer patients and 2 ml freshly-made 0.8% paraformaldyde (PFA) were mixed and incubated for 10 minutes at room temperature. Next, the sample is applied to the microfluidic chip and washed with 1X PBS twice and fixed in 1% (v/v) formaldehyde/PBS. Immediately prior to hybridization, the cells are treated with 200 ul of 0.05% (w/v) pepsin to 1.8 ml of 0.01 N HCl, 4 ml cold 70% (v/v) ethanol, and washed with 1X PBS/MgCl2. FISH probes are denatured at 73°C with 70% formamide for 5 minutes. The probes are then placed on ice. One ml of pre-warmed denature buffer is added to the cells on the microfluidic chip and the manifold is heated by on-board heater (73°C, 2 minutes). The denatured probe is added into the microfluidic chip and incubated for 5 minutes at 73°C and hybridized overnight at 37°C under dark conditions. Her-2 and Chromosome 17 probes from Vysis (Abbott Molecular, Evanston, IL) are used to carry out the experiments. Hybridized cells were washed with 2X SSC/0.1% NP-40 (v/v)) at room temperature, counter-stained with 4’, 6-diamidino-2-phenylindole (DAPI) and sealed.

### mRNA Fluorescence in-situ hybridization (FISH)

Two milliliters of blood samples from metastatic breast cancer patients and 2 ml freshly-made 0.8% paraformaldehyde (PFA) are mixed and incubated for 60 minutes at room temperature. Next, the sample is applied onto the microfluidic chip and washed with 1X PBS twice. Immediately prior to hybridization, the cells are treated with 150 ul of Ready-To-Use Protease #3 (Advanced Cell Diagnostics #310842) for 15 minutes at room temperature and washed with 1X PBS twice. The cells are washed with 70% ethanol and 1X PBS twice. Pan-cytokeratin and CD45 probes (Advanced Cell Diagnostics, CA) are incubated for 10 minutes at 40°C and cooled to room temperature. One ml pre-warmed denature buffer is added to the cells on the microfluidic chip and heated microfluidic chip using on board heater (73°C, 2 minutes). Five drops of the probe are added to the microfluidic chip and incubated overnight at 37°C. The cells on the microfluidic chip are the washed with 1 ml of 2X SSCT twice. Three amplification steps are carried out according to the manufacturer’s instructions (Advanced Cell Diagnostics, CA). Hybridized cells then were counter-stained with 4’, 6-diamidino-2-phenylindole (DAPI) and sealed.

## Results

### Capturing CTCs from patients with metastatic breast, prostate and colorectal cancer

**Fig 2:**
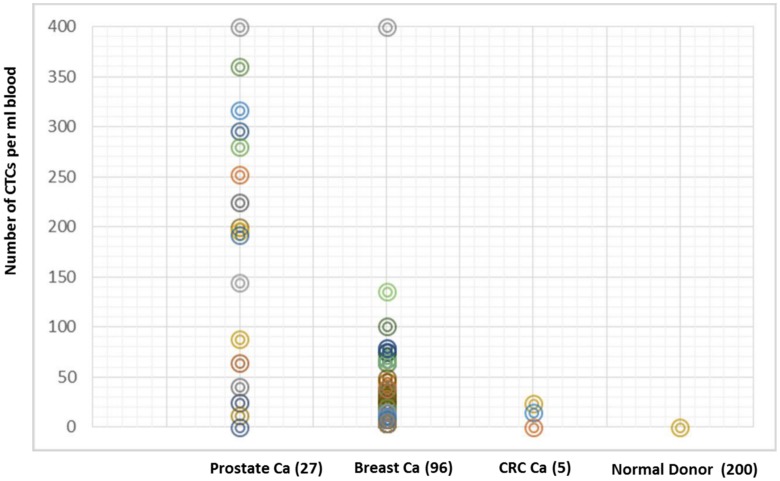
Detection and enumeration of CTCs in blood samples from patients with metastatic breast, prostate and colorectal cancer. Number of CTCs (Y-axis) in 2ml of blood captured by the Celsee PREP 400 system from healthy donors and patients with metastatic breast, prostate and colorectal cancer.

Blood samples from 200 healthy donors and 128 patients with metastatic breast (n = 96), prostate (n = 27) and colorectal cancer (n = 5) were processed using the Celsee PREP 400 system. The Celsee PREP 400 system showed excellent performance characteristics in the detection of CTCs in the patient samples from all 3 cancer types and no detection of any CTC in 200 blood samples from normal donors. CTCs were seen in each of the cancer patient samples, and the number of CTCs per 2 ml blood is shown in Fig 2 (range 1–2,457 cells). No CTC was found in any of the healthy donors (Fig 2).

### Comparison of Celsee PREP assay and CellSearch assay

**Fig 3:**
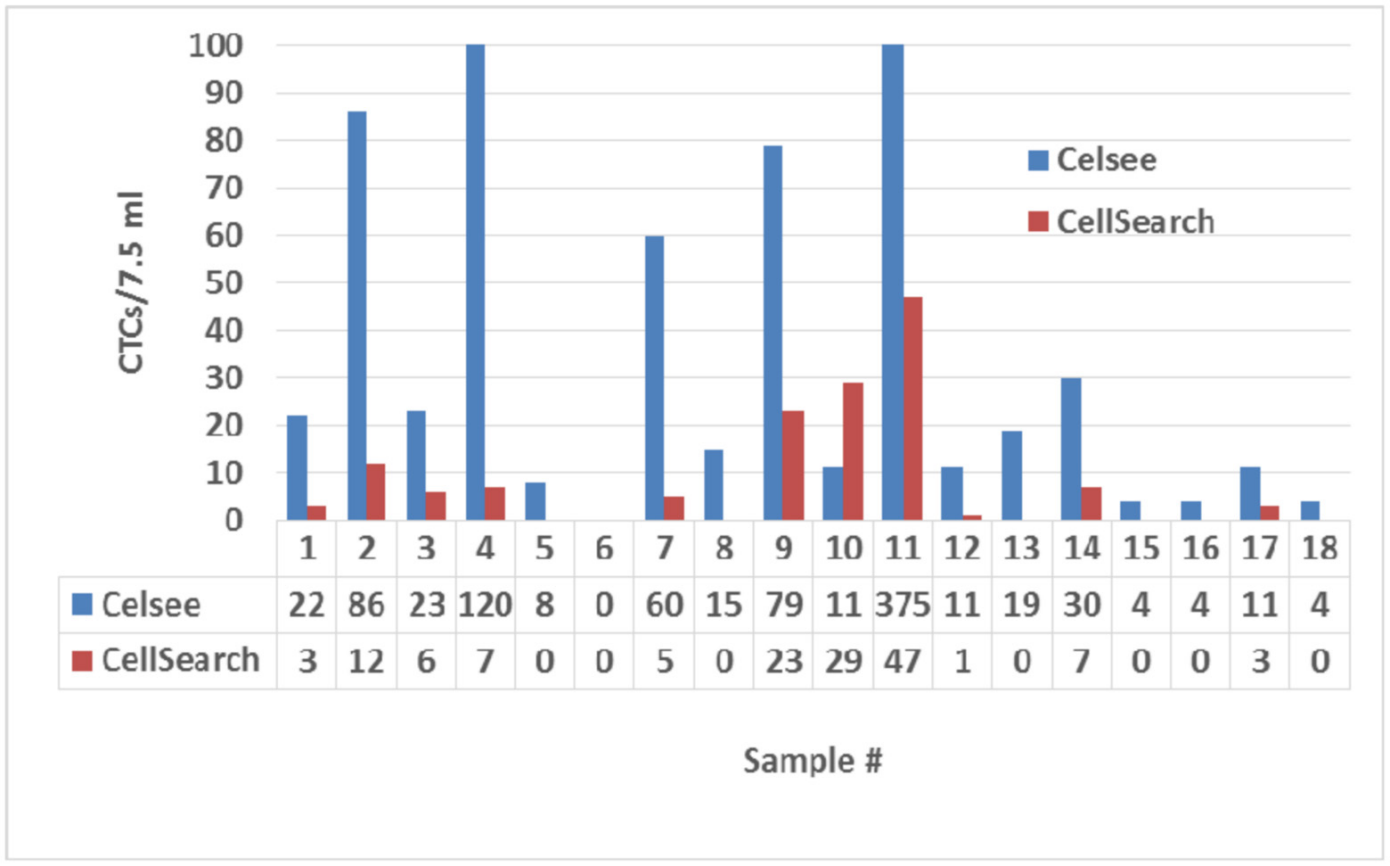
Comparison of CTC counts using the Celsee PREP 400 microfluidic device and the CellSearch system. Number of CTCs measured by either Celsee PREP 400 or CellSearch are standarized to CTCs per 7.5 ml of blood. Twenty samples from patients with metastatic prostate cancer are included in the study.

We compared CTC enumeration values between the Celsee PREP 400 system and the FDA-cleared CellSearch system using 18 blood samples from patients with metastatic prostate cancer. Two tubes of the blood samples were collected from each of the patients at the same time. One tube of blood was processed by the CellSeacrh system according to the manufacturer’s manual (Veridex, Raritan, NJ), and the other tube of blood was processed by the Celsee PREP 400 system. CTCs were detected in 61% (11/18) samples using CellSearch, and 94% of the samples (17/18) by the Celsee PREP 400 system. CTC counts were significantly higher using the Celsee PREP system, implying greater sensitivity for CTC detection (Fig 3).

### Immunostaining of CTCs

**Fig 4:**
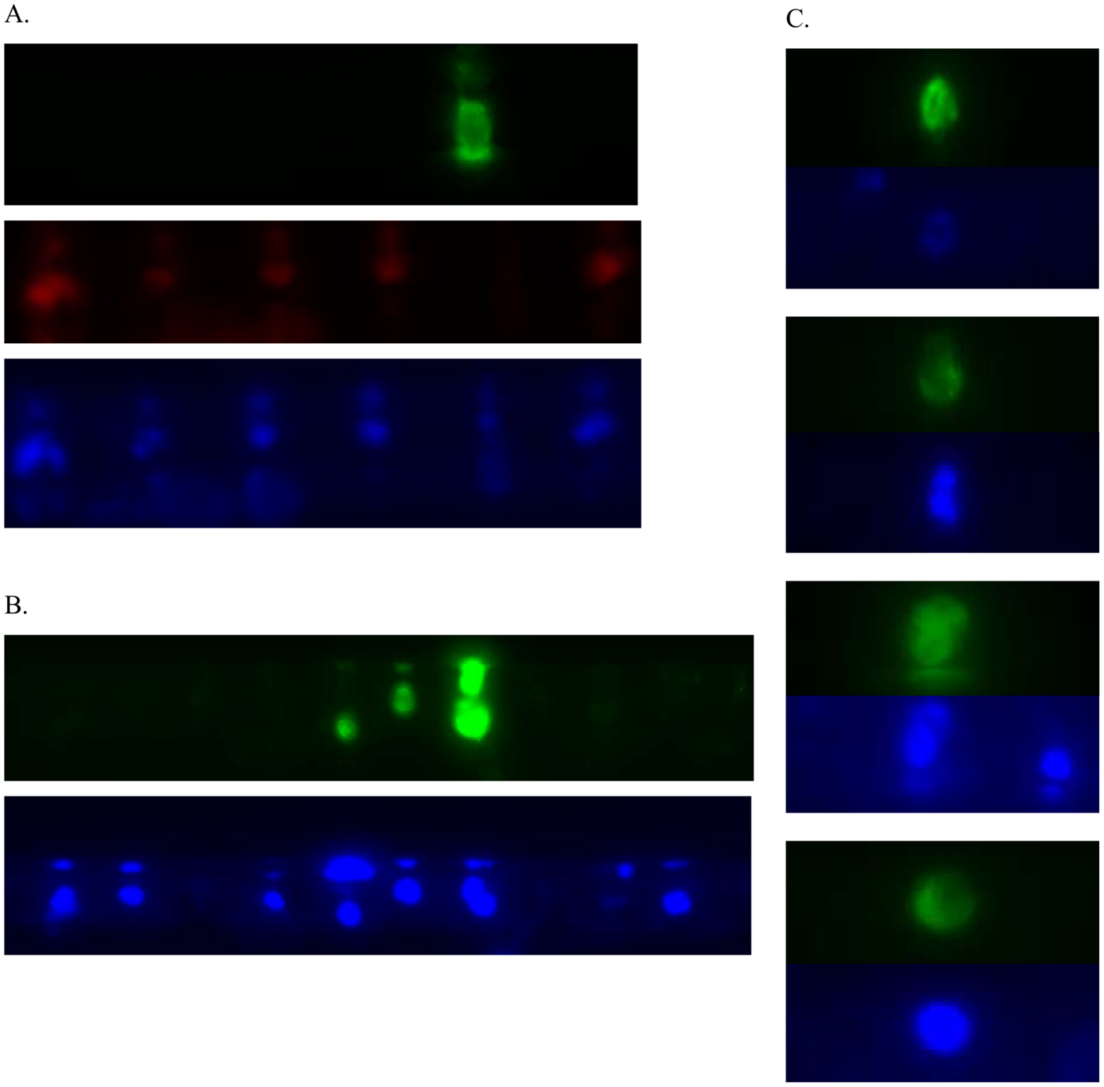
Immunostaining of enriched CTCs from cancer patients. (A). A representative image of staining by DAPI, panCK and CD45 antibodies. CTCs are identified by the following criteria: DAPI+ (blue), panCK+ (green) and CD45- (red). (B) Immunofluorescence staining of enriched cells with anti-vimentin. A representative image of staining for DAPI (blue) and vimentin (green). (C) Immunofluorescence staining of enriched cells with anti-PSA. A representative image of staining for DAPI (blue) and PSA (green).

To characterize the captured cells using additional molecular biomarkers, we performed immunostaining experiments to analyze the expression of epithelial or mesenchymal-specific proteins. Positive staining of PanCK in the captured CTCs were observed, suggesting an epithelial nature of these cells (Fig 4A). In addition, a few selected samples were stained with anti-vimentin antibody. A high level of vimentin expression was observed in some of the captured cells. The detection of vimentin, a specific marker for mesenchymal cells suggested that the Celsee PREP 400 system capture different types of cells (Fig 4B). The vimentin positive CTCs are also larger and different as compared to the PanCK stained CTCs (Fig 4B). In order to confirm that the captured cells are from the tumor, we carried out an experiment of PSA immunostaining of the captured cells from metastatic prostate cancer patients. The result confirmed that the captured cells are PSA positive, nucleated cells suggesting that they are from prostate tumors (Fig 4C). We used CD45 as a marker for leukocyte staining to distinguish background blood cells from the captured cancer cells. Examples of the stained leucocytes are shown in Fig 4. Our results suggested that the Celsee PREP 400 system can capture both epithelial cancer cells as well as other cancer cells. The microchannel device captured a very low level of leukocytes, and these cells were discriminated from CTCs with differential immunostaining using cell type specific antibodies.

### Characterization of CTCs by DNA FISH and mRNA FISH

**Fig 5:**
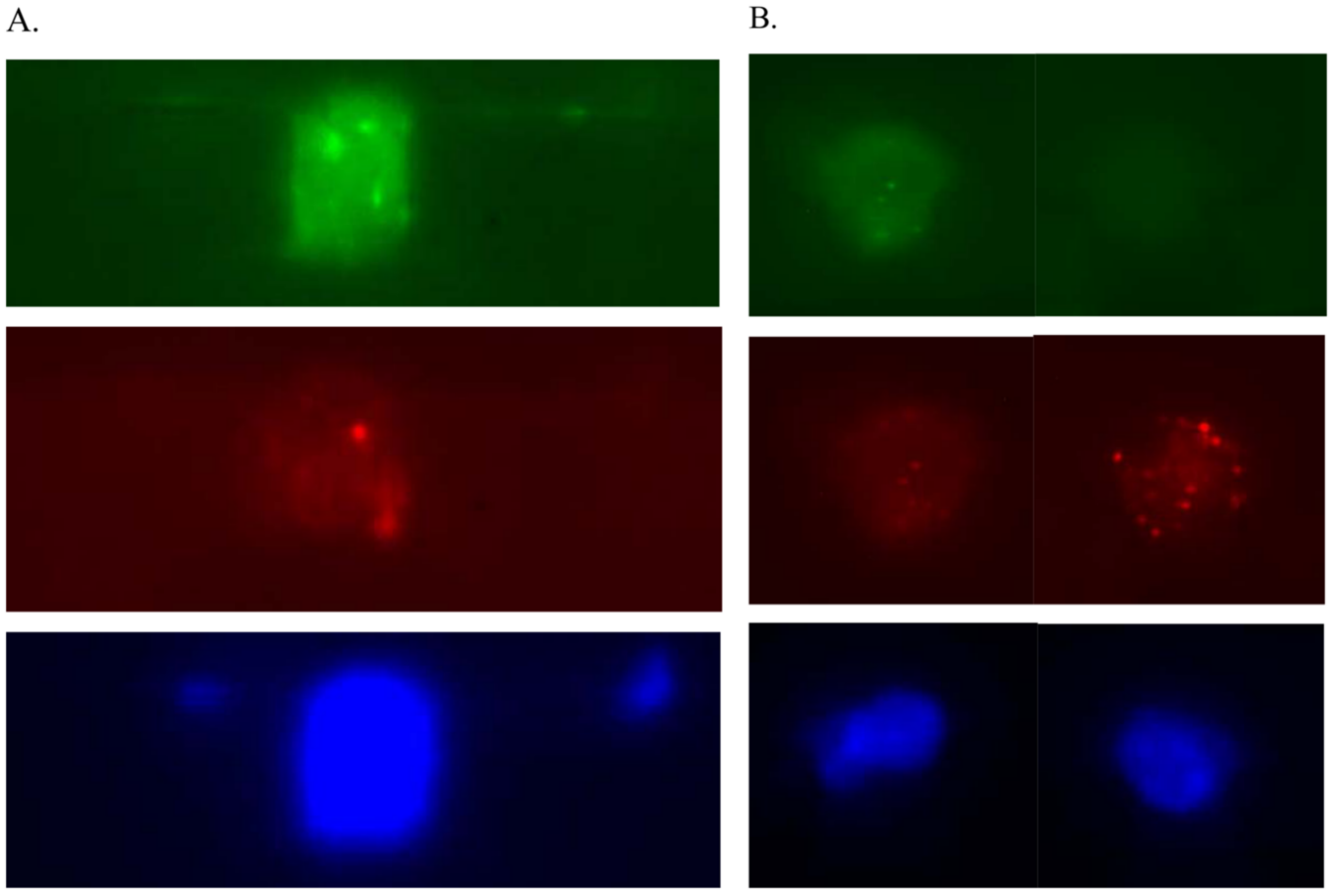
DNA FISH and mRNA FISH analysis of enriched CTCs from metastatic breast cancer patients. (A) DNA FISH of enriched CTCs. A representative image of CTC hybridized to Her-2 (green), Chromosome 17 (red) probes and stained by DAPI (blue). (B) mRNA FISH of enriched CTCs. A representative image of CTC (left panel) and a leukocyte (right panel) hybridized to panCK (green), CD45 (red) probes and stained by DAPI (blue).

We performed an automated DNA FISH assay in CTCs on the Celsee PREP 400 system using the Vysis Her-2 and Chromosome 17 probes (Abbott Molecular, IL) after enumeration and fixation of captured cells from breast cancer patients. The idea here is to use Chr 17 probe as the control (2 copies, 2 dots) and to find cells that has amplified signals with HER-2 probe (> 2 copies, >2 dots). This would suggest that the cell is not only a breast cancer cell, but also a cancer cell with HER-2 amplification (Fig 5A). Similarly, we were able to automate mRNA FISH assay in CTCs on the Celsee PREP 400 system using the PanCK and CD45 probes (Advanced Cell Diagnostics, CA) after enumeration and fixation of captured cells (Fig 5B). In both experiments we compared the automated assays with the manual assays using the manufacturers’ instructions. The automated assays performed as well as the manual assays in pre-treatment, hybridization and post-hybridization washing (data not shown).

## Discussion

We demonstrated that CTCs can be readily captured and further characterized with molecular markers from clinical blood samples of patients with metastatic breast, prostate and colorectal cancer. Using the automated Celsee PREP 400 system, we obtained high definition images of immunostained CTCs (nucleated PanCK+/CD45- cells) in all 3 cancer types tested. Furthermore, we compared the detection and enrichment of CTCs between the Celsee PREP 400 system and the CellSearch system. In 20 prostate cancer patients with paired CTC counts, we demonstrated some levels of concordance in CTC detection by both systems. The Celsee PREP 400 system showed a higher sensitivity than the CellSearch system and detected CTCs from all 20 samples. A possible expalnation could be due to the presence of heterogeneous CTC populations with low or absent EpCAM expression, as these may not be captured by the anti-EpCAM antibody used in the CellSearch system.

There is currently ongoing work to apply single-cell molecular analysis on captured CTCs, and the system described here facilitates the characterization of individual cells. We thus further characterized the captured CTCs using on-device immunostaining of additional antibodies, DNA FISH and mRNA FISH assays. Immunostaining of the CTCs on the device suggested that protein biomarkers can be used to detect differential expression of the captured cells. In addition to PanCK, vimentin was selected for the study because that it has been implicated to be specific for mesenchymal cells. Our results indicate the presence of CD45-/CK- cells that may be more mesenchymal in nature. We also developed DNA and mRNA FISH procedures to observe Her-2 amplification in CTCs of the breast cancer patients and pan-cytokeratin mRNA expression in CTCs. Additionally, although background leukocytes can create a challenge for detecting and analyzing CTCs, the present technology isolates leukocytes in separate microchambers and did not affect the analysis of the captured cells.

Several methods have been reported to capture circulating tumor cells from blood with variable degree of success. Microfluidic approaches offer some advantages but such devices have been so far limited to research tools that lack automation and validation necessary for implementation in a clinical laboratory. A key feature of this platform is the ability to integrate the process of sample preparation, image analysis of CTCs and molecular characterization of the captured cells. Furthermore, the unique design of the microchannels of the chip allows separation of CTCs from the background leukocytes and improve sample purity over other methods that capture cells in aggregates. We are working on recovering single cells from the microfluidic chip for PCR and next-generation sequencing analyses. Progress towards understanding the heterogeneity and the complexity of CTCs in cancer could shed further light on the mechanisms of tumor metastasis and the delineation of tumor cells that are relevant to prognosis and therapy choice. It would also help to overcome some limitations of clinical use of CTC technologies, such as lack of standardization of CTC definition, low sensitivity in detection of CTCs in subsets of patients and high background leukocytes.

Although the comparison study between the Celsee platform and the CellSearch platform, a FDA-cleared platform for CTC capturing suggested that the Celsee platform capture more cells, the study has some limitations because the sample size is relatively small. A more rigorous study needs to be carried out between our platform and CellSearch, especially to demonstrate the cause of discrepant measurements between the 2 platforms. We are proceeding with a clinical trial for the Celsee system (instruments, reagents and software) for clinical use.

In summary, there continues to be a need for simple, non-invasive and inexpensive predictive tests for monitoring and determining response to therapy. Through improved CTC enumeration and molecular characterization it may be possible predict therapeutic response based on a single blood draw. Further development of the presented technology could potentially facilitate personalized treatment strategies and improved outcomes.

## Supporting Information

S1 Table CTC Counts in Patient Blood Samples. (XLSX)
